# Long‐Term Headache Outcome and Radiological Correlates in Patients With Intracranial Hypotension

**DOI:** 10.1111/ene.70051

**Published:** 2025-02-12

**Authors:** Marina Romozzi, Giuseppe Garignano, Antonio Funcis, Renata Martinelli, Marco Rossi, Paolo Calabresi, Catello Vollono, Francesco Signorelli

**Affiliations:** ^1^ Department of Neuroscience Università Cattolica del Sacro Cuore Rome Italy; ^2^ Dipartimento di Neuroscienze, Organi di Senso e Torace Fondazione Policlinico Universitario Agostino Gemelli IRCCS Rome Italy; ^3^ Radiology and Neuroradiology Unit, Dipartimento di Diagnostica per Immagini e Radioterapia Oncologica Fondazione Policlinico Universitario Agostino Gemelli IRCCS Rome Italy; ^4^ Depatment of Neurosurgery, Fondazione Policlinico Universitario Agostino Gemelli IRCCS Università Cattolica del Sacro Cuore Rome Italy; ^5^ Department of Anesthesia and Intensive Care Fondazione Policlinico Universitario Agostino Gemelli IRCCS Rome Italy

**Keywords:** CSF, epidural blood patch, headache, intracranial hypotension, leak

## Abstract

**Background:**

Headache is the most common presenting symptom of intracranial hypotension (IH), and it usually has orthostatic features. However, the outcome of IH and the persistence and characteristics of headache are still overlooked.

**Methods:**

In this cohort study, patients diagnosed with IH in our institute between 2018 and 2024 were included. Demographical and clinical data, headache characteristics, etiology, type of treatment (epidural blood patch (EBP), surgical or conservative), and MRI findings were collected. We conducted follow‐up visits on headache characteristics and the persistence of headache ≥ 12 months of EBP/conservative treatment.

**Results:**

Forty‐five patients with a diagnosis of IH were included (mean age of 53.0 ± 14.9 years); 35 (77.8%) were diagnosed with spontaneous intracranial hypotension (SIH) and 10 (22.2%) with secondary IH. EBP was performed on 22 patients (48.9%). Headache was the most common symptom at presentation, in 38/45 patients (84.4%), with orthostatic features in 32 (71.1%). Forty‐four patients (97.8%) had brain MRI abnormalities. Follow‐up visits were conducted after 31.6 ± 15.7 months; 28/41 (68.3%) patients reported headache during the first 12 months, and 22/41 (53.7%) ≥ 12 months. Headache persistence for ≥ 12 months was significantly lower in patients who received EBP (27.3%) compared to those who did not (63.2%) (*p* = 0.021). Logistic regression showed that receiving EBP was the only factor significantly associated with reduced likelihood of persistent headache for ≥ 12 months (OR = 0.082, 95% CI [0.007,0.903], *p* = 0.041). Radiological features differed significantly between patients with SIH and those with secondary etiologies.

**Conclusion:**

A large proportion of patients with IH continue to experience headache beyond one year; EBP was the only predictor of headache persisting ≥ 12 months.

## 1 Introduction

Headache represents the predominant clinical presentation of intracranial hypotension (IH) [1]. Intracranial hypotension may result from spontaneous spinal cerebrospinal fluid (CSF) leak configuring in spontaneous intracranial hypotension (SIH) or can be secondary to iatrogenic procedures or trauma. Although orthostatic headache is the prototypic manifestation of IH, headache at onset may exhibit different features, such as non‐orthostatic headache, thunderclap headache, or a non‐orthostatic chronic daily headache [2]. Typically, accompanying symptoms include nausea, vomiting, photophobia, and phonophobia. Furthermore, these patients may experience a variety of symptoms, including tinnitus, aural fullness, neck pain, subtle cognitive problems, stiffness, and dizziness [2]. According to the third edition of the International Classification of Headache Disorders (ICHD‐3), SIH is diagnosed when headache has manifested spontaneously and in temporal relation to low CSF pressure (< 60 mm of H20) and/or radiological evidence of CSF leakage [3]. In addition to the clinical symptoms, the qualitative and quantitative typical features of IH in magnetic resonance imaging (MRI) can be useful as a diagnostic cue, however, 1 in 5 patients with SIH may have normal MRI findings [4]. Although the symptoms of IH can be debilitating and significantly impair the quality of life for affected individuals, IH is often an underreported and misdiagnosed disorder [5].

The main therapeutic approaches for spontaneous and secondary IH are conservative treatment and the epidural blood patch (EBP) [6]. Epidural blood patch, a procedure first described in 1960, is a minimally invasive treatment consisting of injection of autologous blood in the epidural space, that usually leads to rapid improvement due to changes in craniospinal CSF mechanics and a sustained effect resulting from tamponade and sealing of the dural defect [6]. This treatment can be a meaningful and often life‐changing intervention and has been reported to be effective in up to 70% when performed early in the disease course in patients with SIH [7]. Prompt intervention is also crucial because CSF loss may result in serious conditions including subdural hematomas requiring evacuation, cognitive decline (frontotemporal brain sagging syndrome), coma, or extensive superficial siderosis [8].

Approximately 40% of patients with SIH require multiple blood patches. Despite treatment, a subset of patients may experience persistent or recurrent symptoms, including headache, dizziness, fatigue, and tinnitus [9, 10]. Several studies have sought to identify predictors of treatment response with inconsistent results, focusing on radiological and clinical aspects, laboratory tests, and technical characteristics of EBP procedure [1, 11, 12].

A meta‐analysis recently performed in our institution to identify factors affecting the outcome of epidural blood patching in SIH reported a good response (defined as complete remission of symptoms within 48 h after the first EBP) in 300 of 500 patients. Among the variables available for analysis, none affected the efficacy of EBP [13].

In such a complex and multifaceted condition, defining a response can be challenging, with variations between studies in determining what constitutes an acceptable response, whether based on clinical or radiological criteria. Headache appears to be a reasonable proxy for assessing response, yet the long‐term characteristics of headache in both SIH and secondary IH, whether patients are treated conservatively or with EBP have not been thoroughly explored.

Therefore, the objective of our study is to investigate the main clinical symptoms of IH after EBP or conservative treatment, in particular, focusing on the long‐term persistence and characteristics of headache and its correlation with radiological features. Finally, the study aimed to compare the headache characteristics and outcomes between spontaneous and secondary IH.

## 2 Methods

### 2.1 Study Design, Population, and Outcome Measures

In this ambispective study, all the patients from our institute between 2018 and 2024 with a diagnosis of IH were included. Patients > 18 years who signed a written informed consent to use their clinical and radiological information for research purposes, with SIH or secondary IH (with documented evidence of surgical intervention, trauma, or lumbar puncture that was temporally linked to the onset of headache or other symptoms) were included. The exclusion criteria were incomplete MRI or clinical data, as well as confounding factors for headache, such as the presence of other neurological, infectious, or hematological conditions that could potentially cause headache or modify its characteristics and not fulfilling the criteria for headache related to IH according to ICHD‐3.

The retrospective part involved collecting, for each patient, demographic data (age, sex), comorbidities, the etiology of IH (spontaneous or secondary to iatrogenic procedures or trauma), and clinical presentation at onset of IH symptoms, including the presence of orthostatic headache (defined as postural headaches that have a baseline headache severity when lying flat of < 2.5 have an onset (or exacerbation) within 90 min of being upright, and an offset to baseline severity within 20 min of recumbency [14]), evaluation of the side of the headache, accompanying symptoms nausea, vomiting, photophobia, phonophobia, monthly headache frequency, pain intensity through the numeric rating scale (NRS), presence of excessive diurnal somnolence and tinnitus. The monthly headache frequency at the presentation was calculated considering all days with headache/time from onset to diagnosis, similarly for the number and type of analgesics per month.

Data on EBP, conservative treatment, or additional treatment were collected. EBP was non‐targeted and performed by a board‐certified neuro‐anesthesiologist (M.R.) using a lumbar midline approach and by injecting a high volume (15–20 mL) of autologous blood as *per* protocol of our institution. Conservative treatment was defined as bed rest, hydration, caffeine, and/or treatment with corticosteroids.

These data were collected from medical charts by two neurologists and headache specialists (A.F. and M.R.) and one neurosurgeon (R.M.) and then confirmed during the face‐to‐face interview conducted at the time of follow‐up to avoid recall bias. During the face‐to‐face interview and clinical evaluation, we not only verified the clinical presentation data that led to medical attention at our institute, serving as a double check for the information retrieved from medical charts but we also focused on collecting detailed information about clinical manifestations and management. The data collected on headache and its characteristics were the following: side of the headache, accompanying symptoms (nausea, vomiting, photophobia, phonophobia), monthly headache frequency, number and type of analgesics per month, concomitant preventive treatment for headache, and NRS score.

Headache related to IH was classified according to ICHD‐3 criteria. Post‐procedural cases were classified according to ICHD‐3 into post‐dural puncture headache (PDPH) and CSF fistula headache. The first is defined as headache occurring within 5 days of a lumbar puncture, caused by CSF leakage through the dural puncture, and the second as orthostatic headache occurring after a procedure or trauma causing a persistent CSF leakage resulting in low intracranial pressure [3]. It was also assessed the presence of a primary headache disorder in the past medical history of the patients. Patients were asked to rate how similar the headache associated with their usual primary headache disorder was to the current headache, using a scale from 0 (very different) to 10 (identical).

Additionally, the presence of headache was assessed ≥ 12 months after treatment, and patients were categorized into two subgroups: those who reported headache after 12 months and those who did not. Data on acute and prophylactic treatments were also collected, with the effectiveness of acute treatment defined as achieving pain relief or pain freedom within two hours.

The study conforms to the ethical guidelines of the 1975 Declaration of Helsinki, as reflected in a priori approval by the institution's human research committee at each participating study site. The study was reported according to the Strengthening the Reporting of Observational Studies in Epidemiology (STROBE) guidelines.

### 2.2 Radiological Measures Assessment

Magnetic resonance imaging findings at baseline (time of first evaluation and pre‐EBP or conservative treatment) were collected and analyzed by a board‐certified neuroradiologist (G.G.). The radiologist was blinded to the clinical presentation and therapeutic approach. To strengthen clinical diagnosis, all the main findings of MRI studies were reviewed and the presence/absence of the following features was evaluated: pachymeningeal enhancement, venous engorgement, suprasellar narrowing (≤ 4 mm), subdural collection, prepontine narrowing (≤ 5 mm), mamillopontine narrowing (≤ 6.5 mm), pituitary gland enlargement, and spinal longitudinal extradural fluid (SLEC). The probability of a spinal CSF leak was calculated with Bern score (recently used as a surrogate marker for clinical severity of IH [15]) a score based on three quantitative and three qualitative radiological signs including: pachymeningeal enhancement (two points), venous engorgement (two points), suprasellar narrowing (≤ 4 mm) (two points), subdural collection (one point), prepontine narrowing (≤ 5 mm) (1 point), and mamillopontine narrowing (≤ 6.5 mm) (one point) [4]. The sum of the points reflects the probability to localize a CSF leak on subsequent myelography: 2 or less points represents low probability, 3–4 points is moderate probability, and 5 or more points are high probability.

### 2.3 Statistical Analysis

Descriptive statistics were used to describe the demographic and clinical features of the sample. Numerical variables were described as mean and standard deviation. Categorical variables were presented as absolute numbers (*n*) and percentages (%). The distribution of each numerical variable was checked with the Shapiro–Wilk Test and parametric or non‐parametric analyses were performed accordingly. Comparisons between two groups (headache persistence after 12 months or not, SIH or secondary IH, orthostatic and non‐orthostatic features at presentation) were performed and compared with the chi‐squared (*χ*2) test and with the Mann–Whitney U test.

A multivariate logistic regression analysis was conducted to examine the associations between various predictors, including EBP and other relevant factors, with the likelihood of experiencing persistent headache lasting more than 12 months. Variables compared in the univariate analysis were entered into a multivariate logistic regression analysis to determine adjusted odds ratios (ORs with corresponding 95% confidence intervals—CI). The model for multivariate analysis was made by choosing variables for the significance in the univariate comparison and for clinical relevance. The statistical significance was set at *p* < 0.05. The study's sample size was not determined based on statistical considerations; all consecutive patients were included. Since few patients had missing data for the variables, no imputation was performed for missing data. All statistical analyses were performed using Statistical Package for Social Science (SPSS) software version 22 (SPSS Inc.).

## 3 Results

### 3.1 Baseline Clinical and MRI Characteristics of the Cohort

**FIGURE 1:**
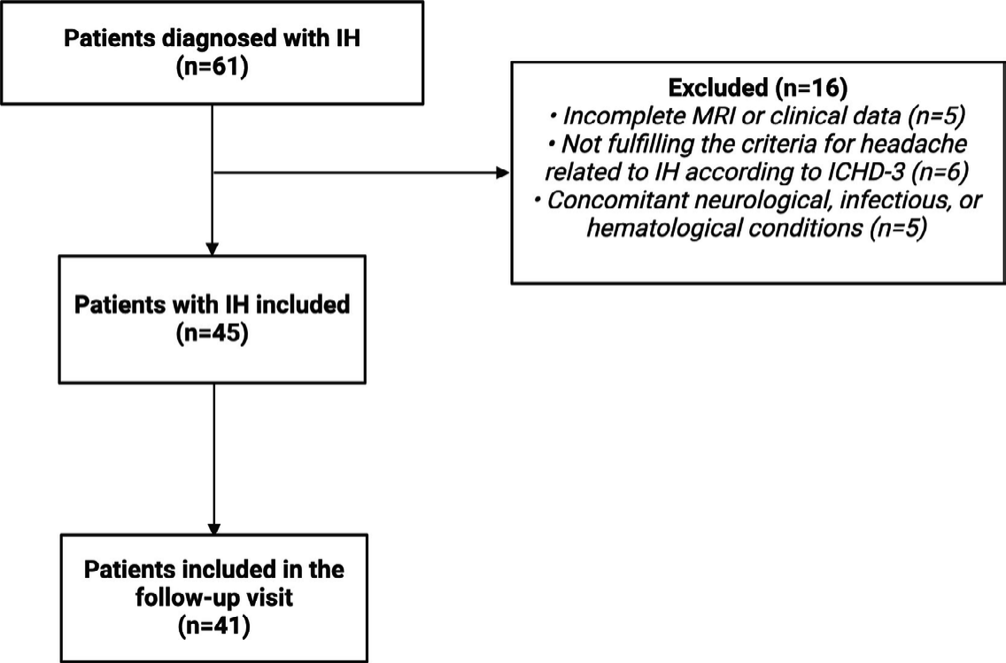
Flowchart of the study. Abbreviations: ICHD‐3, International Classification of Headache Disorders, 3rd edition; IH, intracranial hypotension; MRI: magnetic resonance imaging.

Sixty‐one patients with IH were diagnosed in our Institution from 2018 to 2024. Sixteen patients were excluded. Forty‐five patients with a diagnosis of IH were included in the study, with a mean age of 53.0 ± 14.9 years (28 females, 62.2%). The flowchart of the study is reported in Figure 1. Of these, 35 patients (77.8%) were diagnosed with SIH, and 10 patients (22.2%) had IH secondary to iatrogenic procedures or trauma. The causes included two cases following a lumbar puncture with symptoms occurring within 5 days, two cases after epidural anesthesia within the last 3 months of symptoms start, two cases following microdiscectomy, two cases after steroid infiltration, and one case after epiduroscopy. Three patients with SIH had connective tissue disorder (Marfan syndrome). The mean time from clinical onset to diagnosis was 111.7 ± 229.7 days. Overall, eight patients (17.8%) had a history of a primary headache disorder. Of these eight patients, who were asked to rate the similarity of their headache related to IH to their primary headache on a scale of 0 (very different) to 10 (identical), four rated it as very different (0), two gave it a rating of 2, one rated it as 4, and one rated it as 8. The demographical and clinical features are presented in Table 1.

**TABLE 1:** Demographical and clinical characteristics of the cohort of patients with intracranial hypotension.

	Cohort of patients with IH (*n* = 45)
Age, years (mean ± SD)	53.0 ± 14.9
Sex, F (*n*, %)	28 (62.2)
Etiology, spontaneous (*n*, %)	35 (77.8)
Time from onset to diagnosis, days (mean ± SD)	111.7 ± 229.7
Primary headache disorder (*n*, %)	8 (17.8)
Headache at presentation (*n*, %)	38 (84.4)
Orthostatic headache at presentation (*n*, %)	32 (71.1)
Monthly headache frequency at presentation, days (mean ± SD)	20.1 ± 9.6
NRS at headache presentation (mean ± SD)	8.9 ± 1.3

Abbreviations: EBP, epidural blood patch; F, female; IH, intracranial hypotension; *n*, number; NRS; numeric rating scale; SD, standard deviation.

Headache was the most common symptom at presentation, reported by 38 out of 45 patients (84.4%), with orthostatic features in 32 of those 38 patients (84.2%). Of the 38 patients reporting headache, it was unilateral in 7 patients (18.4%), bilateral in 29 patients, and unilateral and bilateral in 2 out of 38 patients (5.3%). Headache was accompanied by nausea in 22 out of 38 patients (57.9%), vomiting in 18 patients (47.4%), photophobia in 13 patients (34.2%), and phonophobia in 17 patients (44.7%). Additionally, 21 out of 45 patients (46.7%) reported tinnitus, and 13 out of 45 patients (28.9%) reported excessive somnolence. One patient out of 45 (2.2%) reported prominent cognitive and behavioral complaints that fulfilled the criteria for brain‐sagging syndrome [16].

The mean pain intensity at presentation, measured on the NRS, was 8.9 ± 1.3. The monthly headache frequency at presentation was 20.1 ± 9.6 days.

In patients with SIH, the headache fulfilled the clinical criteria of SIH according to the ICHD‐3. Two patients with secondary IH fulfilled the criteria for PDPH, and eight for CSF fistula headache.

All patients except for one (97.8%) in our cohort had brain MRI abnormalities, including the engorgement of venous structures in 21 patients (46.7%), enlargement of the pituitary gland in 17 patients (37.8%), subdural fluid collections in 34 patients (75.6%), enhancement of the pachymeninges in 32 patients (71.1%), suprasellar abnormalities in 25 patients (55.6%), prepontine abnormalities in 8 patients (17.8%), and mamillopontine distance abnormalities in 19 patients (42.2%). The mean Bern score of the cohort was 5.6 ± 2.7.

**FIGURE 2:**
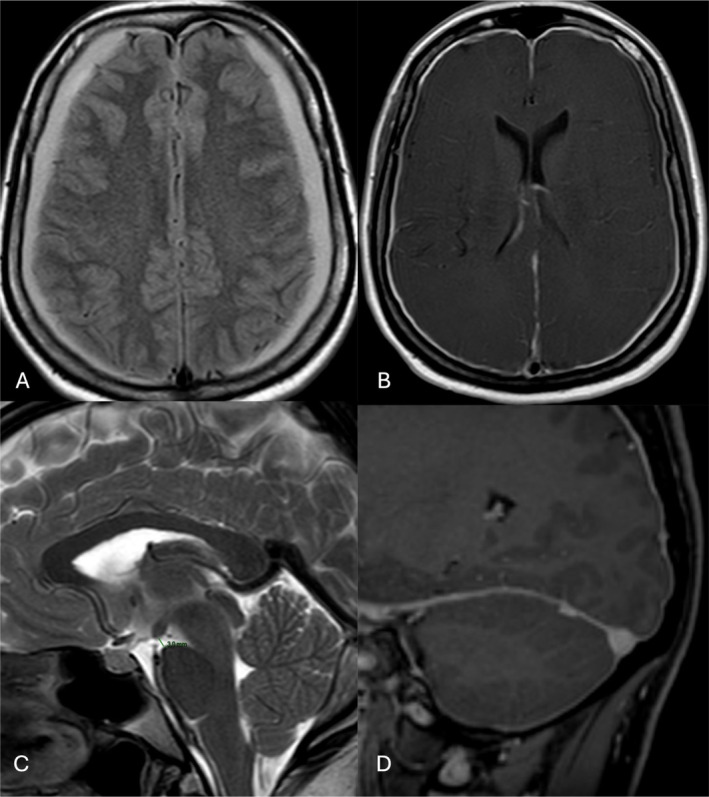
Characteristic brain magnetic resonance imaging (MRI) findings of a patient with spontaneous intracranial hypotension. (A) Axial FLAIR‐weighted image with subdural effusion. (B) Axial T1 after gadolinium injection with pachymeningeal enhancement. (C) Sagittal T2 showing sagging of brainstem with reduced mamillopontine distance (3 mm). (D) Sagittal T1 gadolinium injection with venous distension of the transverse sinus.

Figure 2 reports a prototypical patient of our cohort with spontaneous intracranial hypotension (SIH).

Spinal MRI was available for 35 patients (77.8%). Evidence of SLEC was present in 16 patients (35.6%).

#### 3.1.1 Comparison Between SIH and Secondary IH

There were no statistically significant differences between SIH (*n* = 35) or secondary IH (*n* = 10) in terms of age (*p* = 0.512), sex (*p* = 0.189), time to diagnosis (*p* = 0.129), time to EBP (*p* = 1.0), presence of tinnitus at presentation (*p* = 0.948), history of primary headache disorder (*p* = 0.379), presence of headache at presentation (*p* = 0.805), orthostatic features of the headache (0.263), localization (*p* = 0.323), severity at presentation (*p* = 0.842), monthly headache days (*p* = 0.136), and accompanying symptoms of headache (nausea, *p* = 0.263; vomiting, *p* = 0.841; photophobia, *p* = 0.161 and phonophobia, *p* = 0.858).

Regarding radiological features, secondary IH cases had a significantly higher prevalence of suprasellar narrowing (*p* = 0.012) and increased venous engorgement (*p* = 0.033). In contrast, the SIH group showed a higher number of patients with subdural collections (p = 0.033). No significant differences were observed between the two groups in terms of mamillopontine narrowing (*p* = 0.634), prepontine narrowing (*p* = 0.070), enlargement of the pituitary gland (*p* = 0.101), and pachymeningeal enhancement (*p* = 0.151) (Table 2).

**TABLE 2:** Radiological features of the cohort of patients with intracranial hypotension.

	SIH (*n* = 35)	Secondary IH (*n* = 10)	*p*
Pachymeningeal enhancement (*n*, %)	25 (71.4)	7 (70.0)	0.151
Venous engorgement (*n*, %)	29 (82.9)	5 (50.0)	**0.033**
Suprasellar narrowing (≤ 4 mm) (*n*, %)	22 (62.9)	3 (30.0)	**0.012**
Mamillopontine narrowing (≤ 6.5 mm) (*n*, %)	15 (42.9)	4 (40.0)	0.634
Enlargement of pituitary gland (*n*, %)	15 (42.9)	2 (20.0)	0.101
Prepontine narrowing (≤ 5 mm) (*n*, %)	8 (22.9%)	0 (0)	0.070
Subdural collection (*n*, %)	29 (82.9)	5 (50.0)	**0.033**
Bern score (mean ± SD)	6.4 ± 2.1	3.7 ± 3.1	**0.005**

Abbreviations: IH, intracranial hypotension; *n*, number; SD, standard deviation; SIH, spontaneous intracranial hypotension; bold values indicate statistical significance.

#### 3.1.2 Comparison Between Patients Reporting Headache With Orthostatic Features and Those Not

There were no significant differences in the clinical, radiological features and management (receiving EBP vs. not) between the group of patients with headache with orthostatic features at presentation versus those not, except for the time from the onset to diagnosis, which was greater in the patients without orthostatic features (292.7 ± 413.1 days vs. 58.1 ± 87.3 days, *p* = 0.03).

### 3.2 Treatment

Epidural blood patch was performed on 22 out of 45 patients (48.9%). No patients had undergone EBP before their initial evaluation at our institution. Three patients (6.7%) underwent a second EBP, and nine patients (20.0%) also underwent the evacuation of a subdural hematoma. In three patients (6.7%), a fistula or meningocele was identified and treated by dural repair with patches and sealants and ligation of the neck of the meningocele with vascular clip. The mean time from onset to EBP was 241.8 ± 608.4 days. Eleven out of 45 patients (24.4%) underwent conservative treatment with intravenous fluids and/or corticosteroids and/or bed rest. Twelve patients (26.7%) underwent conservative treatment with bed rest and hydration. Regarding symptomatic treatment for the headache at presentation (*n* = 38), 100% of the patients had used symptomatic treatment. The headache at presentation was treated with nonsteroidal anti‐inflammatory drugs (NSAIDs) in 31 patients (81.6%) and a combination of NSAIDs and caffeine in 8 patients (21.1%); in one case out of 38 (2.8%), triptans were prescribed. Fifteen patients (39.5%) reported that the acute treatment was effective.

### 3.3 Headache Characteristics at Follow‐Up

Out of the 45 patients initially included in the study, one (2.2%) was in critical condition and declined participation in the visit, one (2.2%) was deceased for unknown causes, and two (4.4%) could not be reached. Follow‐up visits were conducted with 41 patients, with a mean follow‐up period of 31.6 ± 15.7 months from the date of diagnosis to the date of follow‐up. None of the patients who have not reported headache at presentation reported headache during the successive follow‐up.

Overall, 28/41 (68.3%) patients reported experiencing headache during the first 12 months after EBP/conservative treatment, with 9 (21.95%) showing orthostatic features. Additionally, 6 out of these 28 patients (21.4%) only reported headache in the first 30 days after EBP/conservative treatment. Among these 28 patients, 19 (67.9%) had bilateral localization of the headache, 3 (10.7%) experienced nausea or vomiting, 5 (17.9%) had photophobia, and 11 (39.3%) had phonophobia. Additionally, 9 (32.1%) reported tinnitus.

Therapeutic management of headache following EBP/conservative treatment included acute treatment with triptans (*n* = 1/28, 3.6%) and other NSAIDs/combination therapy (*n* = 26/28, 92.9%) and prophylactic treatment in 4/28 patients (14.3%), 2 with pregabalin and 2 with amitriptyline.

Headache was reported ≥ 12 months after EBP/conservative treatment in 22 out of 41 patients (53.7%), with a mean monthly frequency of 7.1 ± 7.0 days and a mean use of 5.7 ± 4.7 symptomatic drugs per month.

#### 3.3.1 Comparison of Patients With Persistent Headache ≥ 12 Months and Not

The comparison between patients who reported headache lasting ≥ 12 months after EBP/conservative treatment (*n* = 22) and those who did not (*n* = 16) is presented in Table 3.

**TABLE 3:** Comparison between patients who reported headache lasting ≥ 12 months after epidural blood patch (EBP) or conservative treatment and those who did not.

	Headache ≥ 12 months (*n* = 22)	No headache ≥ 12 months (*n* = 19)	*p*
Age	52.5 ± 14.5	52.4 ± 13.7	0.972*
Sex F (*n*, %)	16 (72.7)	9 (47.4)	0.072°
Etiology, spontaneous (*n*, %)	17 (77.3)	15 (78.9)	0.897°
EBP, (*n*, %)	6 (27.3)	12 (63.2)	**0.021** **°**
Time from onset to diagnosis (mean ± SD)	65.8 ± 95.0	182.4 ± 330.0	0.122*
Primary headache disorder	6 (27.3)	1 (5.3)	0.074°
Orthostatic headache at presentation	17 (77.3)	13 (68.4)	0.524°
Monthly headache frequency at presentation	20.8 ± 10.5	18.1 ± 9.1	0.417*
Monthly headache frequency after EBP/conservative treatment	6.6 ± 7.1	6.0 ± 6.8	0.854*
NRS at headache presentation	8.9 ± 1.5	9.0 ± 1.3	0.832*
NRS after EBP/conservative treatment	6.2 ± 1.7	3.0 ± 3.6	**0.002** *
Bern score	5.3 ± 3.0	6.1 ± 2.3	0.413*

Abbreviations: EBP, epidural blood patch; F, female; *n*, number; NRS, numeric rating scale; SD, standard deviation; * denotes Mann‐Whitney U test; ° denotes Chi‐squared test; bold values indicate statistical significance.

There was a statistically significant difference in the persistence of headache lasting ≥ 12 months between patients who received EBP and those who did not, with rates of 27.3% versus 63.2%, respectively (*p* = 0.021). Additionally, patients with headache lasting ≥ 12 months reported significantly higher residual pain intensity following conservative treatment/EBP (*p* = 0.002). Persistence of headache > 12 months was not correlated to any of the radiological variables examined.

**FIGURE 3:**
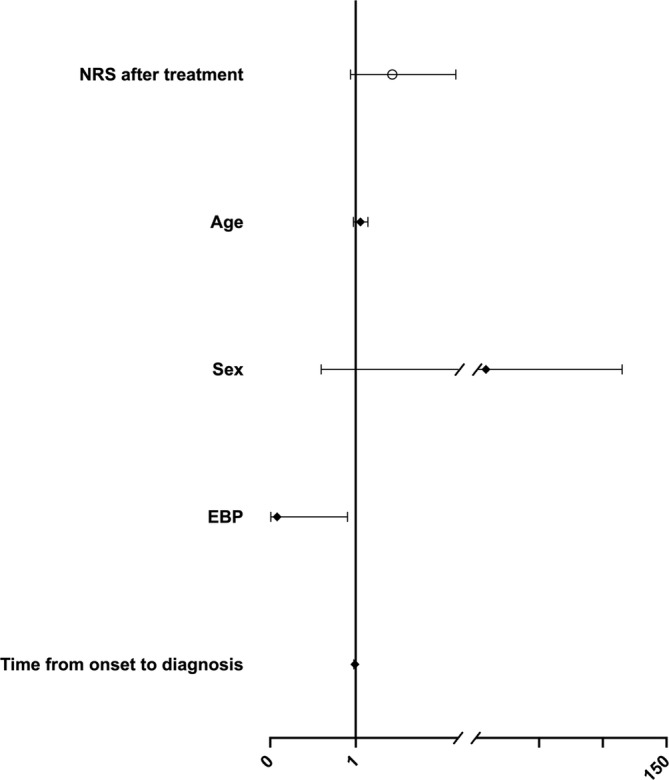
Multivariate analysis with persistent headache ≥ 12 months as a dependent variable. Abbreviations: EBP, epidural blood patch; NRS, numeric rating scale.

Logistic regression analysis showed that receiving an EBP was significantly associated with a reduced likelihood of persistent headache ≥ 12 months (OR = 0.082, 95% CI [0.007, 0.903], *p* = 0.041). Other factors, such as NRS after treatment (OR = 1.427, 95% CI [0.939, 2.171], *p* = 0.096), age at onset (OR = 1.055, 95% CI [0.974, 1.143], *p* = 0.191), sex (OR = 8.293, 95% CI [0.597, 115.134], *p* = 0.115), and time from symptom onset to diagnosis (OR = 0.991, 95% CI [0.978, 1.003], *p* = 0.151), were not statistically significant (Figure 3).

## 4 Discussion

The study results indicate that headache is the most common complaint at the initial presentation of IH (84.4%), with orthostatic features in 71.1% of cases, and we provided a detailed characterization of headache clinical features, accompanying symptoms, and symptomatic and preventive treatment approaches. However, 68% of patients continue to experience headache within the first year following EBP or conservative treatment, with an orthostatic component in 23% of patients, and 53% still have headache beyond one year. This persistence of headache appears to be unrelated to the radiological features observed on MRI at presentation. Regardless of etiology, EBP emerges as the only significant predictor associated with the presence of headache after the first year since the initial presentation.

Headache as the clinical presentation of IH has been thoroughly investigated, especially in association with SIH. In a recent meta‐analysis, headache was the most frequent symptom at SIH presentation, being present in 97% of patients, and was most commonly orthostatic (92%) [1]. However, 3% of patients did not report any headache [1]. The headache location was most commonly diffuse, occipital, or frontal and the most common accompanying symptoms were nausea/vomiting in 54% of the patients and photophobia in 11% of the patients [1]. Similarly, one study of 90 patients with SIH found that orthostatic headache was present in 67 patients (75%), but only in 53 (59%) of these appeared within 15 min of standing; the headache was non‐orthostatic in 22 (24%) and one patient did not report headache [17].

According to the literature and supported by this study, EBP remains the mainstay of treatment, demonstrating a significant advantage over conservative approaches. Recent evidence also supports that EBP should be considered for any patient with orthostatic headaches, even if the brain MRI is negative and the patient does not fully meet the ICHD‐3 criteria for headache attributed to SIH [10, 18].

However, despite treatment, headache may persist for months or even years. It can fluctuate in intensity, sometimes losing or diminishing the orthostatic component and decreasing in severity [19, 20].

In many studies assessing the response to EBP, reported outcomes often vary significantly, with a general reference to “symptom remission” that lacks specificity regarding the symptoms being evaluated. Additionally, there is frequently a predominant focus on the complete remission of orthostatic headache, which may fail to adequately reflect the diverse clinical manifestations of SIH. This is particularly relevant for patients with a wide range of symptoms and headache that is not necessarily orthostatic, especially in the long term [9, 21]. In recent studies, patient‐reported outcomes have only just begun to be considered in the evaluation of treatment response or prognosis [10].

Nevertheless, several studies have sought to identify predictors of response to EBP focusing on radiological findings, laboratory tests, and technical aspects of the blood patching procedure, such as the volume of autologous blood injected and the delivery method (targeted vs. non‐targeted) [1, 11, 12]. Levi et al. reported that the only MRI predictors of a favorable outcome were an iter location > 2 mm below the incisural line and a pontomesencephalic angle < 40° [11]. Similarly, Wu et al. identified three major predictors of response to targeted EBP: the length of anterior epidural CSF collection, the severity of the diencephalic‐mesencephalic deformity, and the volume of blood injected [21]. Lastly, normal MRI findings were also associated with poor outcomes in patients with SIH [22].

In the ICHD‐3 classification, headache attributed to low CSF pressure includes not only the category of headache attributed to SIH but also two additional categories: PDPH and CSF fistula headache [3].

In our study, patients with secondary IH due to lumbar puncture, trauma, or iatrogenic procedures were clinically similar in terms of demographic characteristics, time to diagnosis, headache features, accompanying symptoms, and severity. However, their radiological characteristics differed significantly. Specifically, the secondary IH group had a significantly higher prevalence of suprasellar narrowing and increased venous engorgement, while the SIH group exhibited a greater number of patients with subdural collections. The literature comparing radiological features between SIH and secondary IH remains limited [23]. However, our findings should be interpreted with caution due to the small sample size of the secondary IH subgroup. Additionally, although the time from onset to diagnosis or EBP does not significantly differ between the two subgroups, radiological findings in patients with SIH may evolve over time and present sequentially [24].

Post‐dural puncture headache has been extensively studied, and recent consensus guidelines have recommended the use of EBP in these patients, particularly when PDPH is refractory to conservative treatment and significantly impairs daily activities, or in cases where PDPH is associated with severe neurological symptoms such as hearing loss and cranial neuropathies [25].

On the other hand, CSF fistula headache, typically described in patients who have undergone surgery for spinal column conditions (such as disc herniation or spinal canal stenosis caused by herniated discs and/or degenerative changes), is a less characterized entity. This condition often raises significant diagnostic challenges, and is frequently unrecognized for several reasons. One contributing factor is that headache due to CSF fistula can be part of post‐traumatic symptoms following brain or spinal injury, with symptoms potentially emerging months after the initial trauma. Additionally, this type of headache may not exhibit a typical orthostatic nature or may go undetected if the patient is bedridden.

This study has several strengths. It includes patients with extended follow‐up periods, assessed both prospectively and retrospectively, with interviews conducted by a headache specialist to ensure accurate diagnosis according to ICHD‐3 criteria. Additionally, a board‐certified radiologist reviewed all imaging exams. Furthermore, most studies focus primarily on SIH rather than on the prognosis of patients and the comparisons between SIH and secondary forms of IH. However, the retrospective nature of the study has intrinsic limitations, including recall bias and skewed data collection, as the data were not originally gathered with research as the primary focus. Another limitation is the relatively small sample size, which limits the ability to draw definitive conclusions about the significance of imaging findings and their clinical correlations. However, this limitation is partially expected due to the rarity of the disease. Additionally, the precise localization of a CSF leak requires the use of myelographic techniques.

## 5 Conclusion

Despite treatment with EBP or conservative measures, a significant proportion of patients continue to experience headache beyond one year. In patients with IH, EBP was the only predictor of headache persisting beyond 12 months, regardless of whether the IH was spontaneous or iatrogenic. No MRI features at the initial presentation predicted long‐term headache persistence. Although secondary IH cases present clinically similar to SIH, their radiological findings differ significantly.

## Author Contributions

**Marina Romozzi:** writing – original draft, writing – review and editing, project administration, formal analysis, validation, visualization, methodology, investigation. **Giuseppe Garignano:** investigation, writing – original draft, validation, visualization, formal analysis, project administration. **Antonio Funcis:** investigation, conceptualization, visualization, methodology. **Renata Martinelli:** investigation, conceptualization, visualization. **Marco Rossi:** supervision, methodology. **Catello Vollono:** methodology, validation, visualization, writing – original draft, supervision. **Francesco Signorelli:** funding acquisition, writing – original draft, methodology, validation, visualization, writing – review and editing, project administration, formal analysis, data curation, supervision.

## Ethics Statement

The study conforms to the ethical guidelines of the 1975 Declaration of Helsinki, as reflected in a priori approval by the institution's human research committee at each participating study site. All patients signed an informed consent to use their clinical and radiological information for research purposes.

## Conflicts of Interest

The authors declare no conflicts of interest.

## Data Availability

Data supporting the findings in the present study are reported in the article and in the supporting materials. The raw data collected and analyzed are available from the corresponding author on reasonable request.
